# Crystal Structure of the Apo-Form of NADPH-Dependent Thioredoxin Reductase from a Methane-Producing Archaeon

**DOI:** 10.3390/antiox7110166

**Published:** 2018-11-17

**Authors:** Rubén M. Buey, Ruth A. Schmitz, Bob B. Buchanan, Monica Balsera

**Affiliations:** 1 Metabolic Engineering Group. Dpto. Microbiología y Genética. Universidad de Salamanca, 37007 Salamanca, Spain; ruben.martinez@usal.es; 2 Institut für Allgemeine Mikrobiologie, Christian-Albrechts-Universität Kiel, 24118 Kiel, Germany; rschmitz@ifam.uni-kiel.de; 3 Department of Plant & Microbial Biology, University of California, 94720 Berkeley CA, USA; view@berkeley.edu; 4 Instituto de Recursos Naturales y Agrobiología de Salamanca (IRNASA-CSIC), 37008 Salamanca, Spain

**Keywords:** redox active site, thioredoxin, disulfide, flavin, NADPH, X-ray crystallography, SAXS, methanoarchaea

## Abstract

The redox regulation of proteins via reversible dithiol/disulfide exchange reactions involves the thioredoxin system, which is composed of a reductant, a thioredoxin reductase (TR), and thioredoxin (Trx). In the pyridine nucleotide-dependent Trx reduction pathway, reducing equivalents, typically from reduced nicotinamide adenine dinucleotide phosphate (NADPH), are transferred from NADPH-TR (NTR) to Trx and, in turn, to target proteins, thus resulting in the reversible modification of the structural and functional properties of the targets. NTR enzymes contain three functional sites: an NADPH binding pocket, a non-covalently bound flavin cofactor, and a redox-active disulfide in the form of CxxC. With the aim of increasing our knowledge of the thioredoxin system in archaea, we here report the high-resolution crystal structure of NTR from the methane-generating organism *Methanosarcina mazei* strain Gö1 (MmNTR) at 2.6 Å resolution. Based on the crystals presently described, MmNTR assumes an overall fold that is nearly identical to the archetypal fold of authentic NTRs; however, surprisingly, we observed no electron density for flavin adenine dinucleotide (FAD) despite the well-defined and conserved FAD-binding cavity in the folded module. Remarkably, the dimers of the apo-protein within the crystal were different from those observed by small angle X-ray scattering (SAXS) for the holo-protein, suggesting that the binding of the flavin cofactor does not require major protein structural rearrangements. Rather, binding results in the stabilization of essential parts of the structure, such as those involved in dimer stabilization. Altogether, this structure represents the example of an apo-form of an NTR that yields important insight into the effects of the cofactor on protein folding.

## 1. Introduction

Thioredoxins (Trxs) participate in dithiol/disulfide exchange reactions that effect structural and functional changes in target proteins via reversible change in the redox state of selected cysteine (Cys) residues. Canonical members of the Trx family contain the common amino acid sequence motif WCGPC in the active site with two strictly conserved Cys that target proteins capable of reversibly forming a disulfide bridge. The Cys are reduced to the sulfhydryl form by a Trx reductase (TR) [1]. In many cases, TRs are dependent on reduced nicotinamide adenine dinucleotide phosphate (NADPH) (NADPH-dependent TR or NTR). Based on phylogenetics, protein structure, and molecular mechanism, two groups of NTRs are recognized: (i) Bacteria, plants, lower eukaryotes, and archaea contain low-molecular weight (LMW)-NTRs of about 35 kDa with a non-covalently bound flavin adenine dinucleotide (FAD) cofactor. These enzymes accept reducing equivalents from NADPH and transfer them to a redox-active disulfide motif (CxxC) that subsequently reduces Trx to its sulfhydryl form in a dithiol–disulfide exchange reaction. These transfer reactions involve a large conformational rearrangement within the enzyme [2]; (ii) High-molecular-weight (HMW)-NTRs are enzymes of about 55 kDa present in eukaryotes that have developed an alternate mechanism for the transfer of reducing equivalents to Trx. Also homodimers with a non-covalently bound FAD, these enzymes are fitted with a third redox-active motif that contains either a Cys or selenocysteine at a mobile C-terminal tail. They have the capacity to transfer reducing equivalents from the CxxC motif to the disulfide in Trx [3].

The structural dynamics and functional mechanisms of NTRs have been extensively studied in bacteria and eukaryotes. In archaea, functional studies of the Trx system are available for several species, including *Aeropyrum pernix* [4], *Pyrococcus horikoshii* [5], *Methanosarcina acetivorans* [6,7], and *Sulfolobus solfataricus* [8]. The archaeal NADPH-linked Trx system is biochemically similar to that of bacteria. However, dissimilarities were detected in *Methanocaldococcus jannaschii* [9] and *Thermoplasma acidophilum* [10], where the thioredoxin system (TS) functions independently of NADPH. Here, the coenzyme F420 provides reducing equivalents for *M. jannaschii* TR (MjTR). *T. acidophilum* TR (TaTR), by contrast, is linked to NADH pyridine [10,11].

The present study was conducted (i) to apply in silico analysis for identifying members of the TS in the mesophilic *Methanosarcina mazei,* and (ii) to determine the X-ray crystal structure of its NTR. *M. mazei* is an anaerobic ecologically important methanoarchaeon with a broad substrate spectrum, able to generate methane using H_2_ and CO_2_, methanol, or methylamines as well as acetate as an energy and a carbon source [12,13]. It represents a model organism for the methanoarchaea.

## 2. Materials and Methods

### 2.1. MmNTR Protein Production and Purification 

The full-length NTR of *M. mazei* strain Gö1 (MM2353) was cloned into pET28a, resulting in a cleavable N-terminal 6xHis-tagged fusion protein. Protein was produced in BL21pRIL *Escherichia coli* cells upon induction with 0.2 mM of isopropyl β-D-1-thiogalactopyranoside (IPTG) at 22 °C. Cells were disrupted by sonication in 20 mM Tris-HCl buffer, pH 7.6, containing 300 mM NaCl, 10% glycerol, and 1 mM phenylmethylsulfonyl fluoride. The crude extract was clarified by centrifugation at 40,000× *g* for 1 h. The enzyme was purified using nickel-nitriloacetic acid (Ni-NTA) chromatography (GE Healthcare) and digested with thrombin (EMD Chemicals, San Diego, CA, USA) in buffer, 20 mM Tris-HCl pH 8, 150 mM NaCl, and 2 mM CaCl_2_, at room temperature overnight. The last step of purification involved gel filtration chromatography (Sephacryl S-300) using 10 mM Tris-HCl pH 7.6, 100 mM NaCl, and 2 mM 2-mercaptoethanol buffer. For protein molecular weight estimation by size exclusion chromatography, the column was calibrated with the Gel Filtration Standard mixture from Bio-Rad.

### 2.2. Protein Crystallization, Data Collection, and Structure Solution

One microliter of a 15 mg/mL protein solution in buffer, 10 mM Tris-HCl pH 8 and 50 mM NaCl, was mixed with 1 µL of a solution containing 0.1 M sodium acetate, pH 4.8, and 1 M ammonium chloride. Protein crystals appeared after 4 days of incubation at 25 °C on a sitting drop crystallization plate. Crystals were transferred to a solution containing Paratone Oil before flash freezing. Diffraction data were collected at a wavelength of 1 Å on the X06DA beamline of the Swiss Light Source synchrotron (SLS, Villigen, Switzerland). The diffraction data were processed and scaled using XDS and XSCALE programs [14]. The MmNTR structure was determined using an approach integrating molecular replacement using Phaser [15] and automated model building. Initially, a homology model of the NADPH-binding domain was used as search tool. The template was generated by FFAS03 server [16] based on the thioredoxin reductase structure of *Thermoplasma acidophilum* (Protein Data Bank (PDB) code 3CTY) [10], freed of non-conserved loops and side chains. Phenix.autobuild [17] was then used for automated model building of the FAD-binding domain. The final structure was refined using the Phenix crystallographic software suite [17], alternating with visual inspection of the electron density maps and manual modeling with Coot [18]. Data collection and refinement statistics are summarized in Table S1. The structures were rendered using Pymol [19]. Atomic coordinates and crystallographic structure factors have been deposited in the PDB under the accession code 4ZN0.

### 2.3. SAXS

SAXS data were collected at the B21 beamline at the Diamond Light Source using an online SEC-HPLC system coupled SAXS setup. In line SEC-SAXS was performed using an Agilent 1200 HPLC system equipped with a 2.4 mL Superdex 200 (GE Healthcare Life Sciences, Amersham, UK) column. Forty-five microliters of a protein sample at 5 mg/mL were loaded onto the size exclusion column equilibrated in 20 mM Tris-HCl pH 7.6, 150 mM NaCl, and 2 mM DTT. Data were collected all along the chromatogram at 3 s per frame, and recorded on a Pilatus 2M detector with a fixed camera length of 4.014 m, covering a scattering vector (q) range from 0.0032 to 0.38 Å^−1^. The X-ray wavelength was set at 1.0 Å, equivalent to 12.4 keV. The scattering data were analyzed using the programs Chromixs [20] and Crysol [21] of the ATSAS package [22].

### 2.4. The Protein Sequence Analysis and Structure Modeling

Homolog protein sequences were retrieved from the National Center for Biotechnology Information’s database using the Blastp program [23]. Protein multiple sequence alignments were performed with ClustalX [24]. Structural homology models were generated using Phyre2 [25] using SaNTR (PDB code 4GCM) as a template (100% confidence, 45% identity; 99% coverage).

## 3. Results

### 3.1. Description of the M. Mazei Trx System

**Figure 1:**
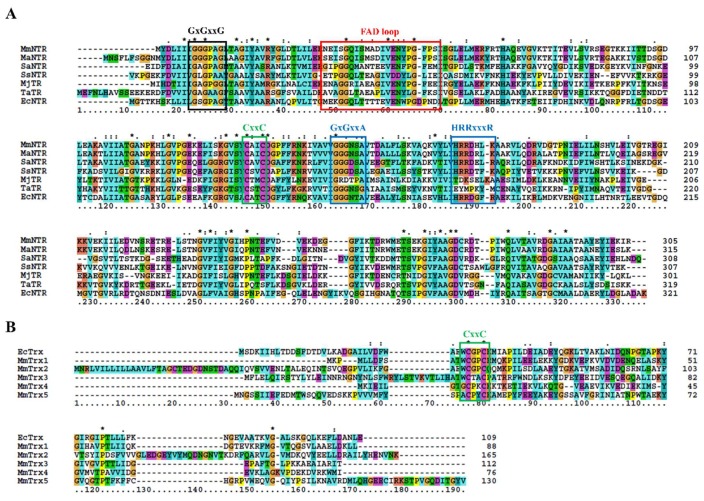
Protein multiple sequence alignment of selected NADPH-dependent Trx reductase (NTR) (**A**) and Trx (**B**). The positions of the conserved residues involved in FAD- and NADPH-binding are marked in panel (**A**) in black and blue, respectively; the region implicated in FAD coordination is depicted in red (see also Figure 3A below). The CxxC redox-active site motifs are shown in green in (**A**,**B**). Asterisks and column colors are assigned according to the ClustalX program’s standard parameters. The ruler below indicates the amino acid position in the alignment. On the right, the number of amino acids for each Trx is stated. The figure includes the amino acid sequences from (**A**) *Methanosarcina mazei* NTR (MmNTR), *Methanosarcina acetivorans* NTR (MaNTR), *Staphylococcus aureus* NTR (SaNTR), *Sulfolobus solfataricus* NTR (SsNTR), *Methanocaldococcus jannaschii* TR (MjTR), *Thermoplasma acidophilum* TR (TaTR), *Escherichia coli* NTR (EcNTR); and, (**B**) *E. coli* Trx (EcTrx), and five Trx sequences from *M. mazei* (MmTrx1–MmTrx5).

The sequence analysis of the genome of *M. mazei* Gö1 revealed an open-reading frame (Locus tag MM_RS12200) that encoded a protein annotated as NADPH-dependent thioredoxin reductase (MmNTR). The amino acid sequence of the NTR-translated gene contained linear motifs for the binding of FAD (GGGPAG) and NADPH (GGGNSA and HRRDHLK) and a CxxC redox motif (Figure 1A), which are typically observed in NTRs [26]. A Blast search [23] of the non-redundant protein database using the MmNTR sequence as input revealed closest amino acid sequence similarity to low-molecular weight NTRs (Figure 1A). A comparison with the NTR enzyme from *M. acetivorans* C2A NTR (MaNTR) showed 83% identity and 92% similarity at the protein sequence level. Two other archaeal TR proteins have been included in the sequence analysis as they are of interest: the TR of *M. jannaschii* (MjTR) that employs F420 as an electron donor [9], and the TR of *T. acidophilum* (TaTR), which was initially described as a pyridine nucleotide-independent TR [10] and has recently been shown to display activity with NADH (but not NADPH) as an electron donor [11]. The MjTR and TaTR sequences lack the motifs necessary for NADPH coordination (the HRRxxxR motif in Figure 1A). Thereby, according to the protein sequence comparison, MmNTR is classified as a canonical LMW-NTR.

Thioredoxins are described as proteins of about 88 amino acids with the canonical motif WCGPC [27]. Using EcTrx as input in Blast homology searches [23], it was found that *M. mazei* contains two Trxs, herein named MmTrx1 (MM_RS02345) and MmTrx2 (MM_RS05160) (Table 1 and Figure 1B). MmTrx1 has a shorter sequence than canonical Trxs at its N-terminus, and its functional annotation awaits further investigations. The MmTrx2 amino acid sequence contains several insertions that provide the protein with additional structural elements compared to EcTrx; like the *M. acetivorans* homolog [6] (Table 1), the MmTrx2 gene is located upstream of the gene encoding the membranous CcdA oxidoreductase [28], suggesting a functional relation.

A sequence analysis of the *M. mazei* Gö1 genome also identified atypical Trxs with varying active sites. NTR was placed adjacent to the gene MM_RS12205 that encoded a predicted Trx protein, herein called MmTrx3, with a WCTAC redox motif (Table 1 and Figure 1B); MmTrx3 is a homolog of Trx7 from *M. acetivorans* (MaTrx7) that has been biochemically demonstrated to be reduced by an NTR [6]. Other atypical Trxs include MM_RS11655 (MmTrx4) and MM_RS10780 (MmTrx5) with the non-canonical catalytic motifs GCPKC and ACPYC (Table 1 and Figure 1B). Their respective homologs in *M. acetivorans* (MaTrx5 and MaTrx1) failed to display insulin disulfide reductase activity and are of unknown function [7]. Recently, a ferredoxin:disulfide reductase (FDR) has been demonstrated to reduce MaTrx5 [29]. FDRs are Fe-S enzymes present in a number of archaea and bacteria that are related to the plant-type ferredoxin:thioredoxin reductase catalytic subunit (FTRc) [30]. Apparently, the NTR and FDR systems are both functional in *Methanosarcina* [29,31,32].

### 3.2. Purification and Structural Features of MmNTR

**Figure 2:**
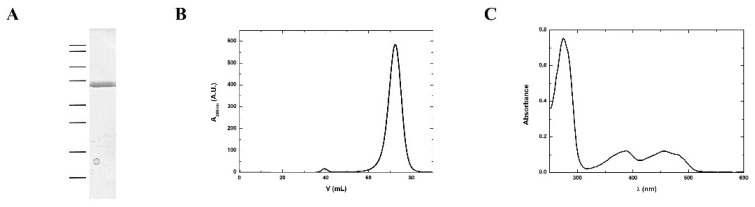
MmNTR was heterologously expressed in *E. coli*, and purified by affinity and size exclusion chromatography. (**A**) A Coomasie-stained SDS-PAGE image of purified MmNTR. The molecular weight markers are indicated on the left (100, 75, 50, 37, 25, 20, 15, and 10 kDa); (**B**) The size exclusion chromatography (Sephacryl S300) elution profile for MmNTR; (**C**) The UV-visible absorption spectrum of MmNTR in buffer, 10 mM Tris-HCl pH 7.6, 100 mM NaCl, and 2 mM 2-mercaptoethanol.

According to the protein sequence, MmNTR has a theoretical molecular weight of 35 kDa. A homogenous sample of MmNTR protein was prepared using the protocol described in the Materials and Methods section. The purified MmNTR migrated as a single band with an apparent molecular mass of 35 kDa in sodium dodecyl sulfate-polyacrilamide gel electrophoresis (SDS-PAGE) (Figure 2A). The purified MmNTR eluted as a single peak during gel filtration chromatography. The deduced molecular mass of 70 kDa indicates that MmNTR is a homodimer in solution (Figure 2B). The protein displayed a yellow color, and showed an absorption spectrum typical of a flavoprotein with peaks at about 274, 388, and 456 nm (Figure 2C).

### 3.3. Apo-MmNTR is a Homodimer Composed of Two Domains

**Figure 3:**
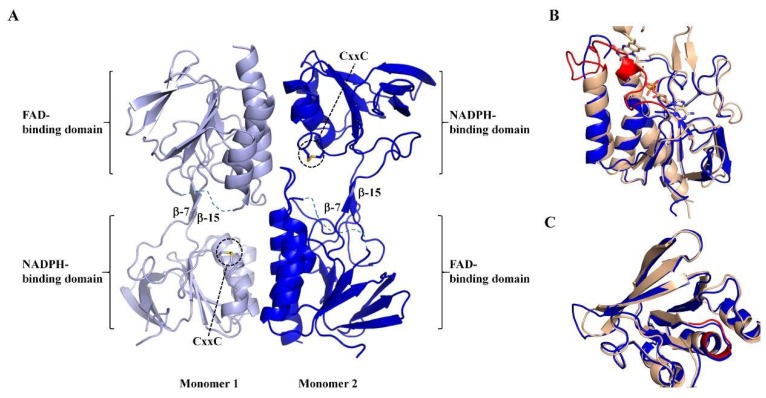
The crystal structure of MmNTR shown in a cartoon representation. (**A**) Monomers of MmNTR homodimers fold into two domains: The FAD-binding domain and the NADPH-binding domain. The Cys of the CxxC motif at the NADPH-binding domain are shown in sticks. The two domains are connected by two beta-strands (β-7 and β-15) that facilitate the conformational flexibility of the molecule. The redox-active disulfide faces inwards, where it would be accessible to reduction by the flavin, and the NADPH-binding site exposed to the solvent. The missing parts of the final model are indicated by dotted lines. (**B**,**C**) Fold comparison of FAD- and NADPH-binding domains, respectively, of MmNTR (in blue) and SaNTR (PDB code 4GCM, in brown). No electron density of FAD was found at the expected cofactor-binding pocket in MmNTR, despite the well-defined and conserved FAD-binding cavity in the folded module. The FAD from SaNTR is drawn as sticks. The FAD-loop and NADPH-binding motif are colored in red.

Protein crystals of MmNTR were obtained, and the X-ray structure was solved and refined to a resolution of 2.6 Å (Table S1). The X-ray structure shows that MmNTR is a homodimer, in agreement with the gel filtration data, with a structural organization that is analogous to other LMW-NTRs. Monomers consist of two Rossmann-fold domains that functionally correspond to FAD- and NADPH-binding domains [33] (Figure 3A). The FAD-binding domain is formed by two discontinuous segments of the polypeptide chain (residues 1–111 and 245–307 in MmNTR), and adopts a three-layer β/β/α-fold with a central four-stranded β-sheet flanked by a three-stranded β-sheet and three parallel α-helices. The NADPH-binding domain (residues 116–240) consists of a four-stranded β-sheet with three parallel α-helices and a three-stranded β-sheet at its flanks. The NADPH-binding domain is inserted into the FAD-binding domain via two antiparallel strands, β-7 (residues 112–114) and β-15 (residues 242–244) (Figure 3A). The redox motif CxxC (residues 132–136) is located on helix- α3 within the NADPH domain, with the two Cys forming a disulfide bridge in the crystal (Figure 3A).

Remarkably, no electron density of FAD or any other molecule was found at the presumed cofactor-binding pocket. The MmNTR structure thus represents the apo-form of the flavoprotein. Attempts to obtain crystals of the FAD-containing enzyme, including the addition of FAD to the crystallization drop, were unsuccessful. A search of structural homologues of the FAD domain of MmNTR using the Dali server [34] revealed that it is highly similar to the FAD-binding domains of other NTRs despite the absence of the cofactor. The FAD domain of MmNTR superimposes to the FAD domain of NTR from *Staphylococcus aureus* (SaNTR; PDB code 4GCM) with a root mean square deviation value of 1.7 Å (Figure 3B). The largest structural variations were found in a region next to the FAD-binding pocket, the so-called FAD-loop, which encompasses residues Ile37 through Pro55 (Figure 3B, in red), that lacks a defined electron density in the apo-structure and is, therefore, omitted from the final MmNTR model. The region constitutes a loop and a 3(10)-helix that covers the FAD isoalloxazine ring on its *si*-face, and a loop involved in dimerization [33]. Because the majority of FAD-contacting residues are strictly conserved in MmNTR (Figure 1A), a similar FAD binding mode is anticipated for this enzyme.

The NADPH-binding domain has no detectable pyridine nucleotide in the crystal structure, but displays essentially the same fold as the equivalent domain in SaNTR (Figure 3C). The binding site of the pyridine nucleotides in SaNTR includes a turn in a region containing conserved glycine amino acids (Gly152–154; Figure 3C, in red), and is dominated by electrostatic interactions between the cofactor phosphates and a cluster of arginine residues (motif HRRxxxR in Figure 1A). The lining of the residues is similar, implying that the cofactor-binding sites of both enzymes are comparable.

### 3.4. Apo-MmNTR Monomers Adopt a Flavin-Oxidizing Conformation

The NADPH-binding domain is positioned in an orientation with respect to the FAD-binding domain that exposes the NADPH-binding site to the bulk solvent and approaches the CxxC motif over the *re*-face of the isoalloxazine ring of the presumed flavin cofactor. This arrangement corresponds to the flavin-oxidizing (FO) conformation of NTRs that allows for the interaction of the disulfide bridge of the CxxC motif with the isoalloxazine ring of the flavin in a conformation competent for the reduction of the disulfide [35]. The two antiparallel beta-strands connecting the two domains are conserved (Figure 3A), and motion of the NADPH-binding domain relative to the FAD-binding domain is anticipated, as it required for the functional activity of the enzyme during the catalytic cycle.

### 3.5. The Absence of the Flavin Cofactor Compromises Dimer Formation

**Figure 4:**
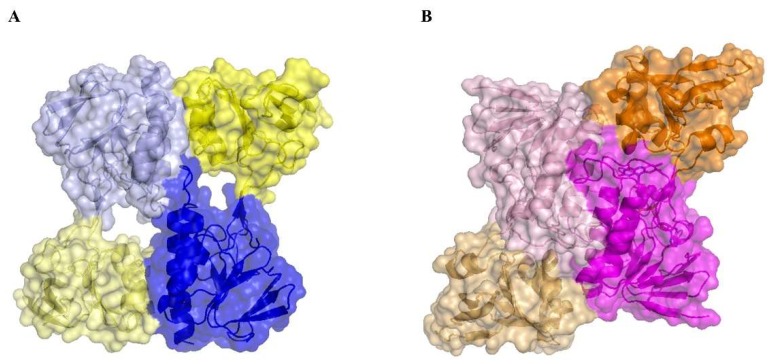
A structural comparison of the homodimer interface in (**A**) MmNTR, with the FAD- and NADPH-binding domains in blue and yellow, respectively; and (**B**) EcNTR (PDB code 1TRB), with the FAD- and NADPH-binding domains in magenta and orange, respectively. The interaction between two monomers within the crystal lattice buries 1696.9 Å^2^ in MmNTR versus 2735.2 Å^2^ in EcNTR of protein surface.

A structural comparison of MmNTR with *Escherichia coli* (EcNTR) homodimers reveals a significant difference at the dimer interface (Figure 4). In EcNTR, two-thirds of the dimer interface is formed by interactions between the FAD-binding domains that involve primarily three α-helices and two loops [36]. By contrast, the FAD-binding domains slide up to the dimer interface of MmNTR with the consequent loss of α-helix contacts. This results in new interactions between the NADPH-binding domain of one monomer with the FAD-binding domain of the opposite monomer (Figure 4 and Figure S1). Subsequently, the two domains in MmNTR are differently positioned each to the other with respect to canonical NTRs. This modification is likely a consequence of the loss of FAD, which results in the structural destabilization of the region comprising residues 36 to 68 that disrupts the dimer interface.

### 3.6. SAXS Reveals a Typical LMW-NTR Conformation of MmNTR

**Figure 5:**
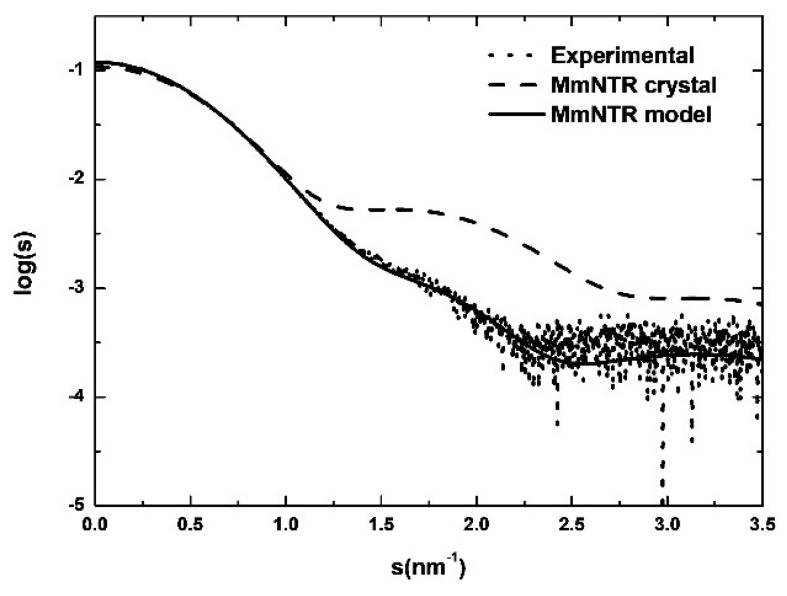
The SAXS study of MmNTR. Experimental SAXS data (dots) overlaid with the theoretical scattering curves that were computed from the crystal structure (dashed line) and the model (continuous line).

We further analyzed the structure of the enzyme using Size-Exclusion Chromatography coupled to with Small Angle X-ray Scattering (SEC-SAXS). As shown in Figure 5, the simulated scattering curve of the crystal structure fits the experimental SAXS data poorly (the curve was computed by Crysol [21]). These discrepancies indicate that the structure of MmNTR in solution differs significantly from the crystal model. Based on these results, a three-dimensional homology model for MmNTR was generated with the modeling server Phyre2 [25] using the crystal structure of SaNTR as a template. The theoretical scattering profiles calculated from the model fit the experimental SAXS curve quite well (Figure 5), and confirm that MmNTR, when bound to FAD, acquires an archetypal FO conformation in solution. These data confirm that MmNTR dimers in solution acquire the same structure as canonical LMW-NTRs.

## 4. Discussion

A genome-wide sequence analysis showed that the composition of the Trx system of *M. mazei* Gö1 is similar to that of other *Methanosarcina* [7], with two Trxs having the canonical WCGPC redox-active motif as well as two Trx-related proteins of diverse features. Two different thiol reductases, NTR and FDR, have been shown to function in the reduction of Trxs in *M. acetivorans*, but not of canonical Trxs. This suggests the presence of other reduction pathways in this type of archaea. The elucidation of alternative reduction systems requires further work; possibilities might include the Fe-S proteins that have been detected in the genomes of *Methanosarcina*, including those that have been related to the catalytic subunit of the FTR protein that is functional in plants [30].

The high-resolution structural analyses of various bacterial NTRs show that LMW-NTRs are homodimers with two Rossmann-fold domains in each subunit [33]. One domain is responsible for FAD coordination (the FAD-binding domain); a second domain carries the CxxC redox-active motif and interacts with NADPH for transferring the reducing equivalents (the NADPH-binding domain). The two domains are joined by two antiparallel beta-strands that coordinate the spatial movement of one domain relative to the other during the catalytic cycle, in the so-called flavin-oxidizing (FO) and flavin-reducing (FR) conformations [35]. The crystal structure obtained in this work shows that MmNTR is a homodimer with two domains per monomer. Despite the lack of a cofactor, MmNTR has a canonical NTR fold. Further, the absence of the flavin results in a locally disordered conformation in a region of the protein that compromises dimer association, thus resulting in abnormal monomer–monomer interactions. 

The protein was crystallized in the FO conformation, with the CxxC motif forming a disulfide bridge placed over the isoalloxazine ring of the assumed FAD. It is anticipated that MmNTR undergoes a general rearrangement that leads to interdomain motion to permit the transfer of reducing equivalents from NADPH to CxxC, and subsequently to Trx.

Our attempts to solve the holo-structure by X-ray crystallography have not been successful so far due to loss of the flavin cofactor during crystallization. Therefore, we performed SAXS studies to obtain structural information on the MmNTR holo-protein, concluding that the conformation of MmNTR in solution is similar to canonical LMW-NTR proteins. The differences between the crystal and the SAXS MmNTR structures might be attributed to the loss of the flavin cofactor, likely during the crystallization process.

## 5. Conclusions

We have presented a description of the thioredoxin system of *M. mazei* Gö1 and the structure of apo-MmNTR obtained by X-ray crystallography and SAXS. Our results show that the structural features of the LMW-NTR family of proteins are well-conserved in the MmNTR structure. The results suggest a primary important role of FAD. The cofactor, which may have been lost during crystallization, appears to function in protein folding since its absence impacts the ability of the enzyme to form the physiological dimer interface. In future work, the three-dimensional structure of the holo-form of the enzyme should provide more accurate data on the conformational organization of the redox-active sites.

## Figures and Tables

**Table 1:** The thioredoxin system composition in *M. mazei* and a comparison with the *M. acetivorans* system.

*M. Mazei* Trx	Gene ID	Catalytic Motif	*M. Acetivorans* Homolog [6]	Reductant	Reduction of Insulin [7]
MmTrx1	MM_RS02345	WCGPC	MaTrx2	unknown	+
MmTrx2	MM_RS05160	WCGPC	MaTrx6	unknown	+
MmTrx3	MM_RS12205	WCTAC	MaTrx7	NTR [6]	+
MmTrx4	MM_RS11655	GCPKC	MaTrx5	unknown	-
MmTrx5	MM_RS10780	ACPYC	MaTrx1	FDR [29]	-

NTR, NADPH-thioredoxin reductase; FDR, ferredoxin:disulfide reductase.

## Supplementary Materials

The following are available online at http://www.mdpi.com/2076-3921/7/11/166/s1, Table S1: Data collection and refinement statistics, Figure S1: Interaction between monomers in MmNTR. The interface is stabilized by hydrophobic interactions, mostly between the side chain of aromatic residues, including Tyr22, Tyr26, Phe161, Tyr301, and Trp264, among others. Hydrogen bonds have also formed between the side chain of the residue Arg142 and the main chain of the residue Arg25, the side chain of Try22 and the main chain of Ile135, as well as a salt bridge between the side chains of the residues Lys164 and Glu302.
