# Supplementary material for: Crystal Structure of the Apo-Form of NADPH-Dependent Thioredoxin Reductase from a Methane-Producing Archaeon
Source: Antioxidants (Basel). 2018 Nov 17;7(11):166. doi: 10.3390/antiox7110166 (PMC6262447; doi:10.3390/antiox7110166)
Supplement: Supplementary file 1 [file antioxidants-07-00166-s001.zip › antioxidants-378180-revised-2-supp for proof/antioxidants-378180-supplementary for proof .docx]

## Table SI. Data collection and refinement statistics.

| **MmNTR*** | |
| --- | --- |
| Wavelength (Å) | 1 |
| Resolution range (Å) | 46.89 - 2.6  (2.693 - 2.6) |
| Space group | C 2 2 21 |
| Unit cell (Å/º) | 117.144 182.5 152.046 90 90 90 |
| Total reflections | 682640 (69703) |
| Unique reflections | 50353 (3650) |
| Multiplicity | 13.6 (14.1) |
| Completeness (%) | 100 (100) |
| Mean I/sigma(I) | 21.49 (2.27) |
| Wilson B-factor | 60.61 |
| R-merge | 0.1016 (1.196) |
| R-meas | 0.1056 (1.241) |
| CC1/2 | 0.999 (0.843) |
| CC* | 1 (0.956) |
| Reflections used in refinement | 50331 (4953) |
| Reflections used for R-free | 2410 (247) |
| R-work | 0.2214 (0.3810) |
| R-free | 0.2410 (0.3835) |
| CC(work) | 0.948 (0.769) |
| CC(free) | 0.931 (0.788) |
| Number of non-hydrogen atoms | 8022 |
| Macromolecules | 7954 |
| Protein residues | 1115 |
| RMS(bonds) | 0.003 |
| RMS(angles) | 0.74 |
| Ramachandran favored (%) | 95 |
| Ramachandran allowed (%) | 4.8 |
| Ramachandran outliers (%) | 0 |
| Rotamer outliers (%) | 3.7 |
| Clashscore | 4.83 |
| Average B-factor | 69.48 |
| macromolecules | 69.70 |
| solvent | 44.00 |
| Number of TLS groups | 12 |
| PDB code | 4zn0 |

Statistics for the highest-resolution shell are shown in parentheses. * Friedel mates were averaged when calculating reflection statistics.
