# Supplementary figures and images for: Crystal Structure of the Apo-Form of NADPH-Dependent Thioredoxin Reductase from a Methane-Producing Archaeon
Source: Antioxidants (Basel). 2018 Nov 17;7(11):166. doi: 10.3390/antiox7110166 (PMC6262447; doi:10.3390/antiox7110166)

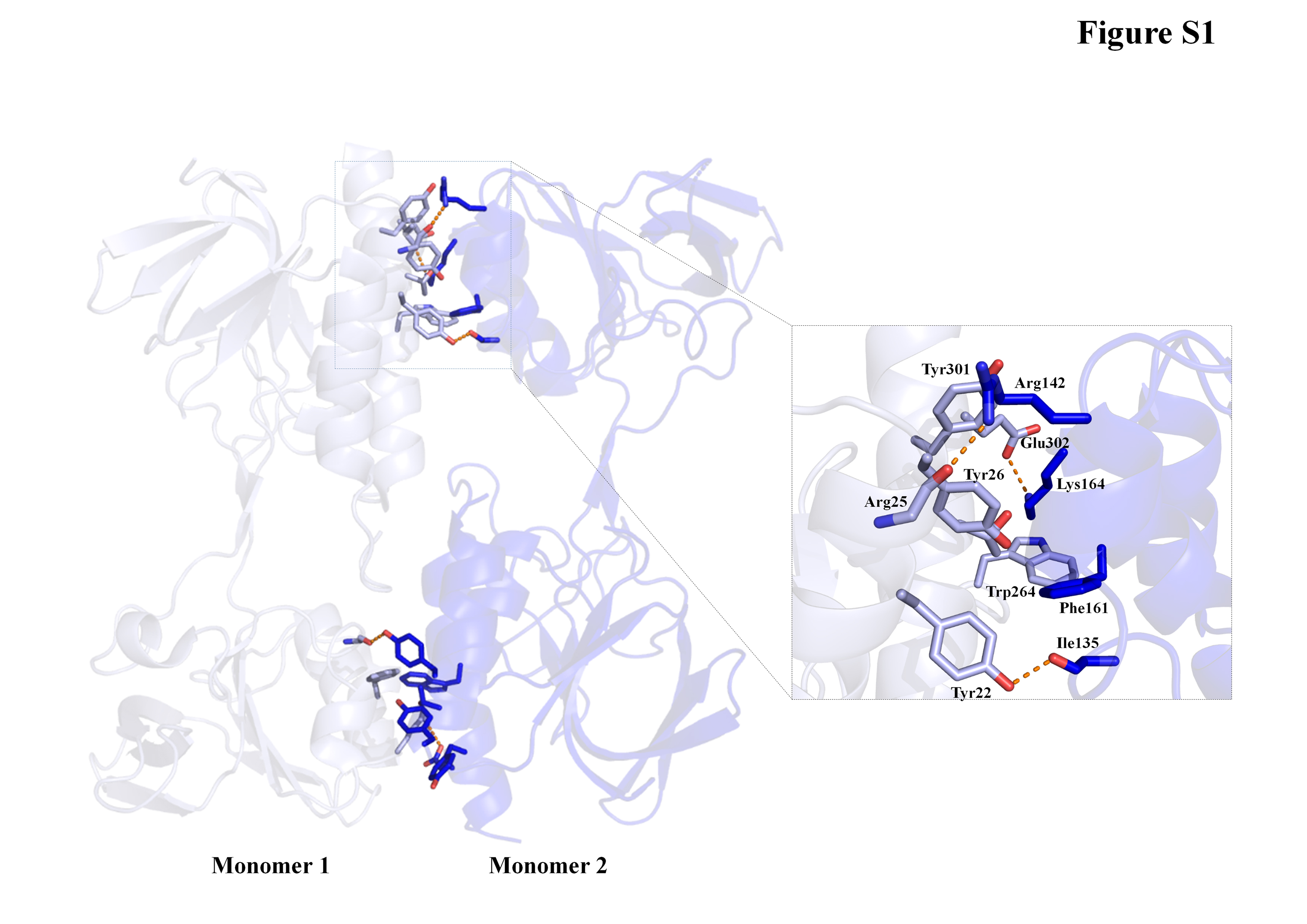

Supplement: Supplementary file 1 [file antioxidants-07-00166-s001.zip › antioxidants-378180-revised-2-supp for proof/FigureS1_BueyAntioxidants.jpg]
